# Disruption and inactivation of the PP2A complex promotes the proliferation and angiogenesis of hemangioma endothelial cells through activating AKT and ERK

**DOI:** 10.18632/oncotarget.4705

**Published:** 2015-07-27

**Authors:** Furong Xie, Xin Bao, Jingshuang Yu, Wantao Chen, Lizhen Wang, Zhiyuan Zhang, Qin Xu

**Affiliations:** ^1^ Department of Oral and Maxillofacial-Head Neck Oncology, Ninth People's Hospital, Shanghai Jiao Tong University School of Medicine, Shanghai Key Laboratory of Stomatology, Shanghai 200011, China; ^2^ Department of Oral Pathology, Ninth People's Hospital, Shanghai Jiao Tong University School of Medicine, Shanghai 200011, China

**Keywords:** hemangioma, PP2A, middle T antigen, endothelial cell

## Abstract

Hemangioma is a benign vascular neoplasm of unknown etiology. In this study, we generated an endothelial-specific PyMT gene-expressing transgenic mouse model that spontaneously develops hemangioma. Based on this transgenic model, a specific binding between PyMT and the core AC dimer of protein phosphatase 2A (PP2A) was verified in hemangioma vascular endothelial cells. The binding between PyMT and the PP2A AC dimer resulted in dissociation of the B subunit from the PP2A complex and inactivation of PP2A phosphatases, which in turn activated AKT and ERK signaling and promoted cell proliferation, migration and angiogenesis *in vitro* and tumorigenesis *in vivo*. Consistent with the *in vitro* findings, decreased PP2A phosphatase activity and disruption of the PP2A heterotrimeric complex were also observed in both primary transgene-positive TG(+) mouse hemangioma endothelial cells (TG(+) HEC cells) and human proliferating phase hemangioma endothelial (human HEC-P) cells, but not in transgene-negative TG(−) mouse normal vascular endothelial cells (TG(−) NEC cells) and human involuting phase hemangioma endothelial (human HEC-I) cells. Further, it was observed that in human hemangioma cells, endoglin could compete with the PP2A/A, C subunits for binding to the PP2A/B subunit, thereby resulting in dissociation of the B subunit from the PP2A complex. Treatment of Tie2/PyMT transgenic mice with the PP2A activator FTY720 significantly delayed the occurrence of hemangioma. Our data provide evidence of a previously unreported anti-proliferation and anti-angiogenesis effect of PP2A in vascular endothelial cells, and show the therapeutic value of PP2A activators in hemangioma.

## INTRODUCTION

Hemangioma, the most common tumor of infancy, is a benign vascular neoplasm resulting from the abnormal proliferation of endothelial cells, with an estimated prevalence of 5 to 10% [1]. These tumors are characterized by a rapid growth phase during the first year of life, followed by slow involution, which may continue until the age of 10–12 years. Despite the progress that has been achieved in the clinical observation and treatment of hemangioma, its etiology remains elusive [2].

To investigate the etiology of this disease, we and others reported a transgenic model of hemangioma that expresses the polyoma middle T antigen (PyMT), the principal oncoprotein of the murine polyomavirus. The transgenic mice were characterized by spontaneous hemangiomas [3, 4]. However, there are still some unresolved problems in this model that limit its application. First, constitutive PyMT overexpression can cause embryonic lethality, resulting in a low frequency of transgenic founders and failure to generate offspring. Second, it is difficult to distinguish between direct and indirect effects of the PyMT protein on endothelial cells during hemangioma formation. Advances in the development of conditional transgenic mice allow type-/tissue-specific transgene expression, overcoming many lethal phenotypes and potentially reflecting direct effects of the transgene on specific tissues/cells [5]. In this study, we generated transgenic mice overexpressing PyMT under the control of the endothelial-specific Tie2 promoter/enhancer [6]. Transgenic mouse lines with a hemangioma phenotype were successfully established. Using this model, we sought to determine the molecular mechanisms underlying the etiology of hemangioma.

Similar to all polyomavirus T antigens, PyMT functions largely via its binding proteins [7]. It was previously reported that PyMT can associate with protein phosphatase 2A (PP2A), a phosphatase known to act as a negative regulator of several survival and proliferation pathways [8]. However, the details of their binding patterns and functions are poorly understood. PP2A is a heterotrimeric holoenzyme composed of a regulatory B subunit associated with a core dimer of a scaffolding A subunit (PP2A/A) and a catalytic C subunit (PP2A/C) [9]. In general, the C subunit always associates with the A subunit *in vivo*. Free C subunits are not found in the cell [10]. The AC core dimer binds to a regulatory B subunit to form the heterotrimeric holoenzyme. At least four families of the regulatory subunits, B (B55), B’ (B56), B’’ (PR72), and B’’’ (PR93), have been identified. The B (B55) subunit is widely distributed, and the other B subunits show differential tissue and developmental distribution [11].

In the present study, based on the Tie2/PyMT transgenic mouse model, a specific binding between PyMT and the PP2A AC core enzyme and subsequent disruption and inactivation of the PP2A complex was observed. Decreased PP2A activity induced downstream AKT and ERK phosphorylation, resulting in rapid growth and increases in the *in vitro* migration and angiogenic ability and *in vivo* tumorigenesis ability of vascular endothelial cells. Disruption of trimeric PP2A holoenzymes and inactivation of PP2A as well as activation of the AKT and ERK pathways were also detected in both primary TG (+) endothelial cells and human proliferating phase hemangioma endothelial cells, which may contribute to hemangioma formation. Moreover, endoglin, a molecule that is specific to newly formed endothelial cells, was found to cause dissociation of the B subunit from the PP2A AC core enzyme in primary human proliferating hemangioma endothelial cells. In addition, treatment with the PP2A activator FTY720 significantly delayed the occurrence of hemangiomas in PyMT transgenic mice. The results of this study provide insights into cellular control mechanisms involved in hemangiomagenesis, showing that disruption and inactivation of the PP2A complex promotes hemangioma formation. Increasing PP2A activity therefore represents a potential approach for hemangioma therapy.

## RESULTS

### Direct expression of the PyMT gene in vascular endothelial cells induced hemangioma formation

**Figure 1:**
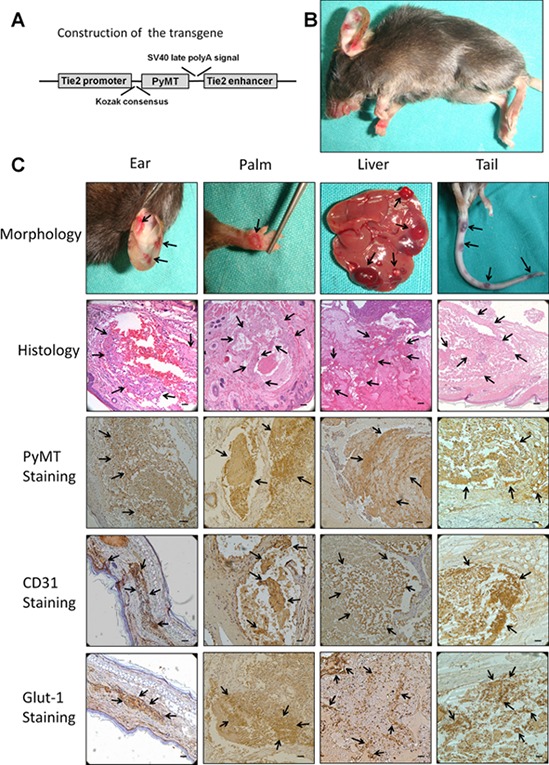
Production and characterization of transgenic mice harboring the Tie2/PyMT gene **A.** Diagram of the Tie2 promoter-driven PyMT transgene. A 2 kb Tie2 promoter is followed by a gene encoding PyMT and a 1.6 kb Tie2 enhancer. **B.** TG(+) mice spontaneously developed hemangiomas in multiple organs (indicated with arrows). **C.** The neoplasms that developed in the ear, palm, liver and tail showed the typical morphological appearance of hemangioma (indicated with arrows). Histological observations showed that the neoplasms were composed of numerous blood vessels lined by plump endothelial cells protruding into the lumen (indicated with arrows). Immunohistochemical staining showed that the endothelial cells of the neoplasms were positive for PyMT, CD31 and Glut-1 (indicated with arrows). Bar = 100 μm

**Figure 2:**
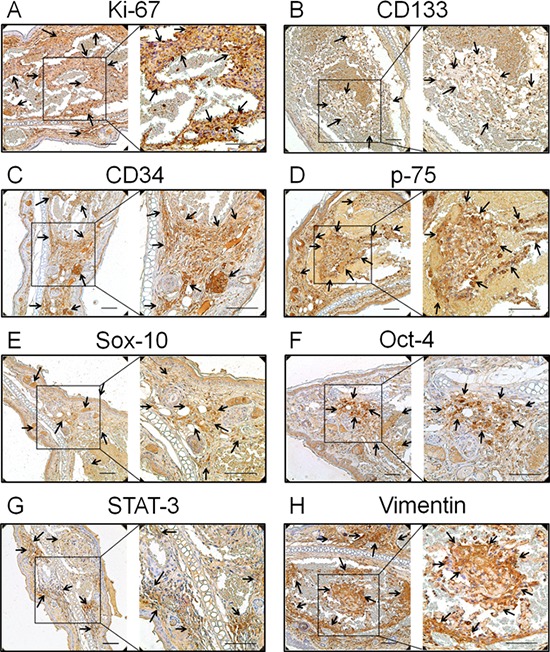
Expression of the proliferation and primitive markers in neoplasms of the transgenic animal **A.** Ki-67, the proliferation marker, **B.** CD133, the haematopoietic stem cell marker, **C.** CD34, the endothelial haematopoietic stem marker, **D.** p75, a cell surface marker of neural crest cells, **E.** Sox-10, the neural crest stem cell marker, **F.** Oct-4, the human embryonic stem cell markers, **G.** STAT-3, another human embryonic stem cell marker, and **H.** vimentin, the mesenchymal stem cell marker, were all positive staining in endothelial cells (indicated with arrows). Bar = 100 μm

To reveal the precise role of the PyMT gene in vascular endothelial cells and to minimize the risk of embryonic lethality in conventional transgenic mice, a strategy for conditional expression of the PyMT gene under the control of the Tie2 promoter/enhancer was adopted (Fig. 1A). TG(+) mice were identified via PCR genotyping, and the phenotypes of these mice were examined. At approximately 45 days of age, all of the TG(+) mice spontaneously developed hemangioma-like neoplasms in multiple organs, such as the skin, tongue and liver (Fig. 1B, C). The neoplasms were composed of numerous blood vessels lined by plump endothelial cells protruding into the lumen, which agrees with the histological structure of hemangioma (Fig. 1C). Immunohistochemical staining showed that the endothelial cells of the neoplasms were positive for both PyMT and CD31 (Fig. 1C). In addition, the positive staining of Glut-1, a marker that differentiates hemangioma from other vascular anomalies and Ki-67, a proliferative marker were also detected in endothelial cells (Fig. 1C, Fig. 2A). However, no obvious revoluting phase was observed in the transgenic mice.

It has been demonstrated that hemangioma is a primitive mesoderm-derived haemogenic endothelium with a neural crest phenotype. Several primitive markers of human hemangioma endothelial cells have been well documented [12, 13]. In this study, the expression of these primitive markers was also investigated in the TG(+) mice. CD133; the haematopoietic stem cell marker, CD34; the endothelial haematopoietic stem marker, p75; a cell surface marker of neural crest cells, Sox-10; the neural crest stem cell marker, Oct-4; STAT-3; the human embryonic stem cell markers and vimentin; the mesenchymal stem cell marker, were all detected in tumor tissues of the transgenic animals (Fig. 2B, 2C, 2D, 2E, 2F, 2G, 2H). This result is consistent with the findings in human hemangiomas, indicating the tumors of this animal model and human hemangiomas have a similar origin.

**Figure 3:**
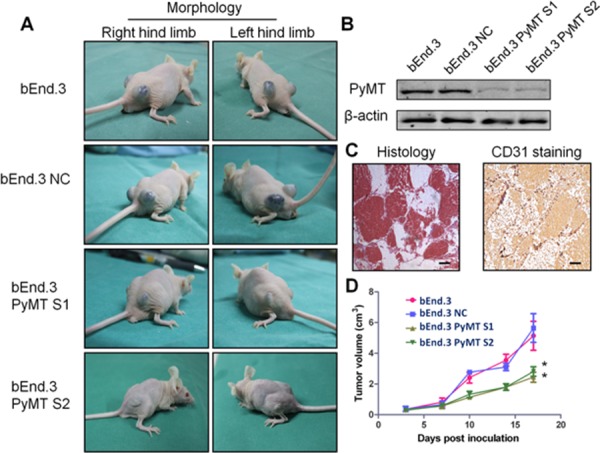
Subcutaneous inoculation of PyMT-expressing endothelial cells induced hemangioma in a murine model **A.** Subcutaneous inoculation of bEnd.3 parental cells, bEnd.3 NC cells (bEnd.3 cells transfected with negative control siRNA) and two PyMT-silenced cell lines (bEnd.3 PyMT S1 and bEnd.3 PyMT S2) into nu/nu mice resulted in the appearance of hemangiomas at the site of injection, and knockdown of the PyMT gene in bEnd.3 cells markedly reduced the ability of bEnd.3 cells to form hemangioma. (*n* = 5/group, two sites per mouse) **B.** Western blotting analysis confirmed PyMT silencing in bEnd.3 cell lines using RNA interference. The resultant stable cells lines were designated bEnd.3 PyMT S1 and bEnd.3 PyMT S2. **C.** Neoplasms from tumor-bearing mice were characterized via histological examination and CD31 staining. Bar = 100 μm **D.** The tumor growth curve showed that silencing PyMT resulted in a decrease in tumor volume. (*n* = 5/group, two sites per mouse, one-way ANOVA) *P < 0.05

Subcutaneous injection of bEnd.3 parental cells, a mouse endothelial cell line immortalized by PyMT, into nu/nu mice resulted in the appearance of hemangiomas within 3 days at site of injection in all animals (Fig. 3A). The hemangiomas were characterized via histological examination and CD31 staining (Fig. 3C). Knockdown of the PyMT gene in bEnd.3 cells (Fig. 3B) markedly reduced the ability of bEnd.3 cells to form hemangiomas (Fig. 3D) (*P* < 0.001).

The formation of hemangiomas in both transgenic and tumor-bearing models indicates that direct expression of the PyMT gene in vascular endothelial cells can trigger the development of hemangioma.

### PyMT inhibits PP2A activity by binding to the core PP2A A/C dimer

**Figure 4:**
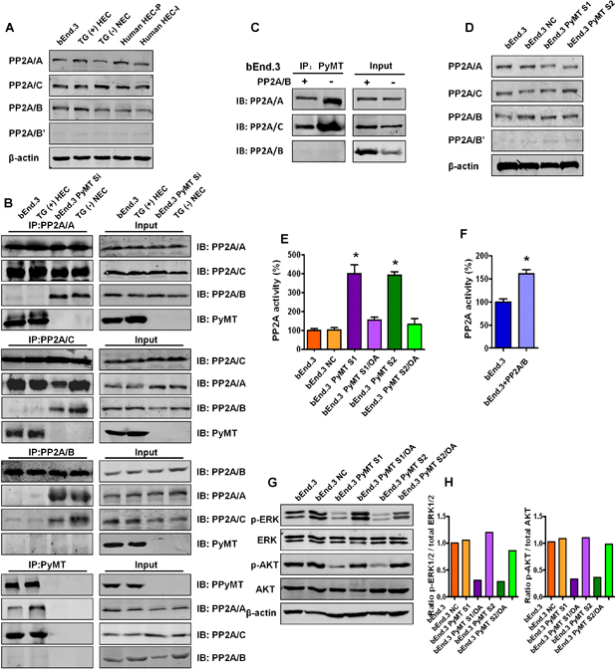
PyMT inhibits PP2A activity by binding to the core PP2A A/C dimer **A.** Expression levels of various PP2A subunits were detected by Western blotting in 5 types of vascular endothelial cells, including bEnd.3 cells, TG(+) HEC cells, TG(−) NEC cells, human HEC-P cell and human HEC-I cells. **B.** The bindings between the PP2A subunits and PyMT were tested via immunoprecipitation (IP) and immunoblot (IB) in both PyMT-expressing cells (bEnd.3 cells, TG(+) HEC cells) and PyMT-deficient cells (bEnd.3 PyMT Si cells, TG(−) NEC cells). Binding between the PP2A/A and PP2A/C subunits was observed in both PyMT-expressing cells and PyMT-deficient cells. In PyMT-deficient cells, the PP2A/B subunit was found to bind to both the PP2A/A and PP2A/C subunits. In PyMT-expressing cells, the PP2A/B subunit showed only a weak or no association with the PP2A/A and PP2A/C subunits, and PyMT was detected only in immunoprecipitates of the PP2A/A and PP2A/C subunits, but not in immunoprecipitates of the PP2A/B subunit. **C.** bEnd.3 cells were transiently transfected with the PP2A/B subunit expression plasmids and cell lysates were subjected to immunoprecipitation with PyMT and probed with anti- PP2A/A, B, C antibody. Competition assay results showed that ectopic expression of the PP2A/B subunit in bEnd.3 cells abolished both the PyMT-PP2A/A and PyMT-PP2A/C bindings. **D.** Expression levels of various PP2A subunits were detected by Western blotting in bEnd.3 cells, bEnd.3 NC cells, bEnd.3 PyMT S1 and bEnd.3 PyMT S2 cells. PyMT silencing did not decrease the protein expression levels of the PP2A subunits. **E.** Phosphatase activity assay results showed that silencing of the PyMT gene led to marked activation of PP2A. Treatment with the PP2A-selective inhibitor OA attenuated the PyMT silencing-induced PP2A activation.(*n* = 3/group, one-way ANOVA) *P < 0.05 **F.** Phosphatase activity assay results showed ectopic expression of the PP2A/B subunit in bEnd.3 cells also led to an increase of PP2A activity. (*n* = 3/group, t test) *P < 0.05 **G.** Western blotting results showed that bEnd.3 cells presented high levels of phosphorylated (active) AKT and ERK, whereas the phosphorylation of both AKT and ERK was down-regulated in PyMT-silenced cells, which could be rescued by OA treatment. **H.** Quantitative analysis of the phosphorylation status of AKT and ERK.

To identify the mechanism through which PyMT induces hemangioma, the bindings between PyMT and PP2A were investigated. We first examined the protein levels of various PP2A subunits in 5 types of vascular endothelial cells, including bEnd.3 cells, primary TG(+) hemangioma endothelial cells, TG(−) normal endothelial cells, primary human proliferating phase and involuting phase hemangioma endothelial cells. As shown in Fig. 4A, the PP2A/A, PP2A/C and PP2A/B subunits were abundantly expressed in these endothelial cells. In contrast, the PP2A/B’ subunit was weakly expressed, and the PP2A/B’’ and PP2A/B’’’ subunits were not detected (data not shown). This result indicates that PP2A/A, PP2A/B and PP2A/C are the major subunits of PP2A in vascular endothelial cells. Based on this result, we narrowed our focus to these subunits to see if PyMT could associate with the subunits. Reciprocal immunoprecipitation was performed in both PyMT-expressing cells (bEnd.3 cells, TG(+) HEC cells) and PyMT-deficient cells (PyMT-silenced bEnd.3 cells (bEnd.3 PyMT Si), TG(−) NEC cells). As shown in Fig. 4B, binding between the PP2A/A and PP2A/C subunits was observed in both PyMT-expressing cells and PyMT-deficient cells, which agrees with previous reports that the PP2A/A subunit always binds to the catalytic C subunit to form a stable AC core dimer *in vivo* [10]. In PyMT-deficient cells, the PP2A/B subunit was found to bind to both the PP2A/A and PP2A/C subunits. In contrast, in PyMT-expressing cells, the PP2A/B subunit showed only a weak or no association with the PP2A/A and PP2A/C subunits, and PyMT was detected only in immunoprecipitates of the PP2A/A and PP2A/C subunits, but not in immunoprecipitates of the PP2A/B subunit. The binding of the PyMT protein to the AC heterodimer in a manner that was mutually exclusive with the regulatory B subunit suggests that expression of PyMT can replace the B subunit in PP2A trimeric complexes.

If PyMT can displace the B subunit, then the PP2A/B subunit might also compete with PyMT for binding to the AC core dimer. We therefore performed the competition assays. bEnd.3 cells were transiently transfected with the PP2A/B plasmid. As shown in Fig. 4C, ectopic expression of the PP2A/B subunit abolished both the PyMT-PP2A/A and PyMT-PP2A/C bindings, which supported the existence of competitive binding between PyMT and the PP2A/B subunit with the core PP2A A/C dimer.

Then, the effects of replacement of the B subunit with PyMT on PP2A were investigated. In both PyMT-expressing cells and PyMT-deficient cells, the protein expression levels of each of the PP2A/A, B and C subunits were not significantly changed following PyMT silencing (Fig. 4D). In contrast, down-regulation of the PyMT gene led to a marked activation of PP2A. PP2A activity was increased nearly four-fold in bEnd.3 Si cells compared with bEnd.3 cells. Treatment with okadaic acid (OA), a PP2A-selective inhibitor, significantly attenuated the PyMT silencing-induced PP2A activation (*P* < 0.001) (Fig. 4E). Moreover, the ectopic expression of the PP2A/B subunit in bEnd.3 cells also led to an increase of PP2A activity (*P* < 0.001) (Fig. 4F). This result indicates that PyMT does not induce alterations in the expression of PP2A, but can inhibit PP2A activity by replacing the B subunit.

### The binding between PyMT and PP2A blocks the dephosphorylation of AKT and ERK

PP2A impacts multiple cell signaling pathways by causing dephosphorylation of signaling proteins. AKT and ERK, two critical signaling molecules, are dephosphorylated by PP2A. To verify the biological impact of the PyMT-induced inhibition of PP2A activity in vascular endothelial cells, we analyzed the phosphorylation status of AKT and ERK in both bEnd.3 parental cells and PyMT-silenced cells. Immunoblot data showed that bEnd.3 cells presented high levels of phosphorylated (active) AKT and ERK. In contrast, silencing of PyMT in bEnd.3 cells resulted in dephosphorylation of both AKT and ERK (Fig. 4G, 4H). This result suggested that PyMT-induced PP2A inactivation enhances AKT and ERK activity by preventing their dephosphorylation. Moreover, OA was shown to reverse PyMT silencing-induced AKT and ERK dephosphorylation, which further supports the role of PyMT in activating AKT and ERK signaling via binding to PP2A.

### PyMT activates AKT and ERK leading to increased cell proliferation, migration and angiogenesis

**Figure 5:**
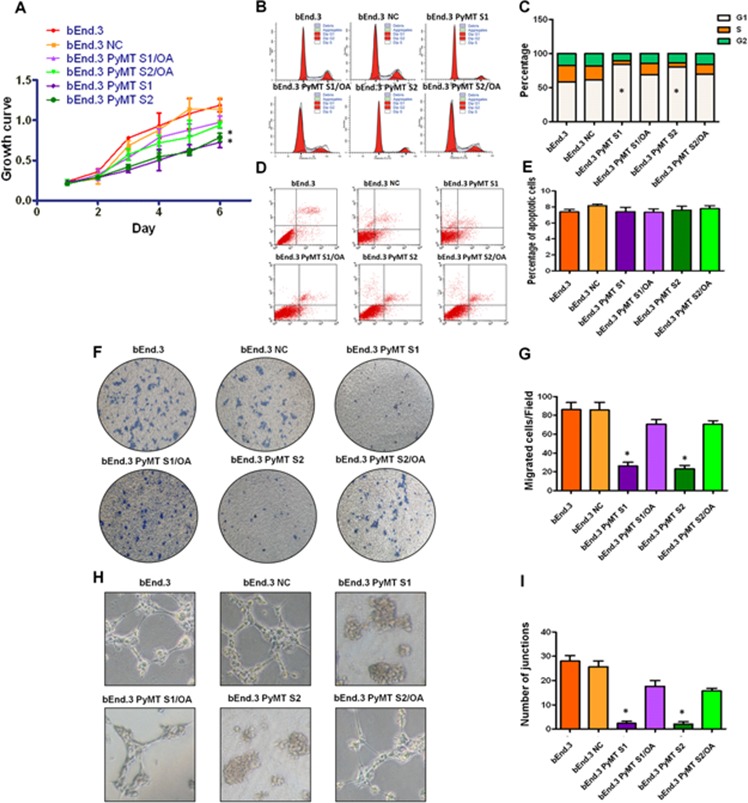
PyMT activates AKT and ERK leading to increased cell proliferation, migration and angiogenesis **A.** bEnd.3 cells displayed higher proliferation than bEnd.3 PyMT Si cells, and bEnd.3 PyMT Si cells regained rapid growth after treatment with OA. (*n* = 3/group, one-way ANOVA) *P < 0.05 **B.** Cell cycles analyzed via FACS. **C.** Obvious G1 cell arrest was observed in bEnd.3 PyMT Si cells compared with bEnd.3 cells, which could also be rescued by OA treatment. (*n* = 3/group, one-way ANOVA) *P < 0.05 **D.** Apoptosis was determined via AnnexinV and PI co-staining. **E.** No significant difference in the number of apoptotic cells was observed between bEnd.3 and bEnd.3 PyMT Si cells. **F.** Transwell assays demonstrated that PyMT silencing in bEnd.3 PyMT Si cells resulted in an approximately 70 percent decrease in migration. OA treatment could rescue this suppression effect. **G.** Quantitative analysis of cell migration. (*n* = 3/group, one-way ANOVA) *P < 0.05 **H.** *In vitro* angiogenesis tube formation assay results showed that bEnd.3 parental cells and bEnd.3 NC cells exhibited an ability to organize and form networks of cords on Matrigel after 48 h of culture, while PyMT-silenced bEnd.3 PyMT Si cells only formed islands of cells, with a few cells migrating out. Treatment with OA partially restored the angiogenic ability of bEnd.3 PyMT Si cells. **I.** Quantitative analysis of junctions number in angiogenesis tube formation assay. (*n* = 3/group, one-way ANOVA) *P < 0.05

AKT and ERK pathway have been implicated to promote cellular growth and proliferation, migration and angiogenesis. We then explored the effects of PyMT-activated AKT and ERK signaling on cell proliferation, cell cycle, cell apoptosis, migration and angiogenesis in both bEnd.3 parental cells and PyMT-silenced cells. Consistent with the phosphorylation status of AKT and ERK, bEnd.3 cells displayed higher proliferation than bEnd.3 PyMT Si cells (*P* < 0.001) (Fig. 5A). Obvious G1 cell arrest was also observed in bEnd.3 PyMT Si cells (*P* < 0.001) (Fig. 5B, 5C). While, no significant difference in the number of apoptotic cells was observed between bEnd.3 and bEnd.3 PyMT Si cells (Fig. 5D, 5E), indicating that PyMT-activated AKT and ERK signaling promotes endothelial cells proliferation, but has no inhibitory effect on apoptosis. Transwell assays demonstrated that PyMT silencing in bEnd.3 Si cells resulted in an approximately 70 percent decrease in migration (*P* < 0.001) (Fig. 5F, 5G). A distinguishing characteristic of vascular endothelial cells is their ability to form vessel/tube-like structures when cultured on three-dimensional extracellular matrices, which reflects their distinct angiogenic ability. Our results indicated that bEnd.3 parental cells formed extensively branched cords of cells on Matrigel after 48 h of culture, whereas PyMT-silenced cells only formed islands of cells, with a few cells migrating out (Fig. 5H, I). In the rescue experiment, treatment of bEnd.3 PyMT Si cells with OA restored AKT and ERK phosphorylation, accompanied by enhanced cell proliferation, cell cycle progression, cell migration and an increased angiogenic ability (Fig. 5A–5I).

### Status of PP2A activity, AKT and ERK phosphorylation and PP2A subunit associations in primary hemangioma endothelial cells

**Figure 6:**
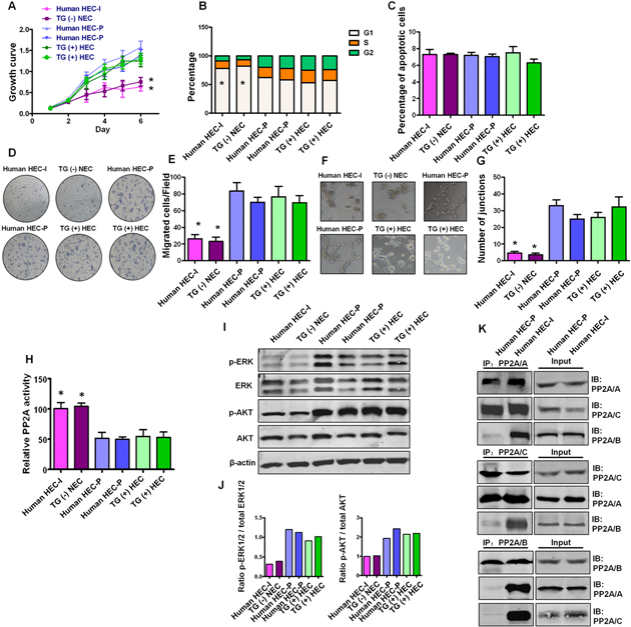
Status of PP2A activity, AKT and ERK phosphorylation and PP2A subunit associations in primary hemangioma endothelial cells **A.** Growth curve shows human HEC-P cells (two lines) and TG(+) HEC cells (two lines) displayed higher proliferation ability than that of human HEC-I cells and TG(−) HEC cells. (*n* = 3/group, one-way ANOVA) *P < 0.05 **B.** Obvious G1 cell arrest was observed in human HEC-I cells and TG(−) HEC cells. (*n* = 3/group, one-way ANOVA) *P < 0.05 **C.** No significant difference in the number of apoptotic cells was observed during these cells. **D.** Transwell assays indicated that human HEC-P cells and TG(+) HEC cells displayed higher migration ability than that of human HEC-I cells and TG(−) HEC cells. **G.** Quantitative analysis of cell migration. (*n* = 3/group, one-way ANOVA) *P < 0.05 **H.** *In vitro* angiogenesis tube formation assay showed that human HEC-P cells and TG(+) HEC cells exhibited higher angiogenic ability than that of human HEC-I cells and TG(−) HEC cells. **I.** Quantitative analysis of junctions number in angiogenesis tube formation assay. (*n* = 3/group, one-way ANOVA) *P < 0.05 **H.** Phosphatase activity assay results showed that inactivation of PP2A was observed in human HEC-P cell lines and TG(+) HEC cell lines compared with human HEC-I cells and TG(−) NEC cells. (*n* = 3/group, one-way ANOVA) *P < 0.05 **I.** Western blotting results showed high levels of phosphorylated AKT and ERK in indicated cell lines. **J.** Quantitative analysis of the phosphorylation status of AKT and ERK. **K.** Immunoprecipitation and immunoblot results indicated the dissociation of the PP2A/B subunit from the PP2A AC core in human HEC-P cells, but not in human HEC-I cells.

To confirm the importance of *in vitro* cell model findings, we examined PP2A activity and the downstream AKT and ERK phosphorylation status as well as PP2A subunit associations in primary transgenic mouse and human hemangioma endothelial cells. Human HEC-P cells, human HEC-I cells, TG(+) HEC cells and TG(−) NEC cells were isolated from human proliferating phase hemangioma specimens, human involuting phase hemangioma specimens, PyMT transgene-positive mice and PyMT transgene-negative mice, respectively. As shown in Fig. 6A–6G, human HEC-P cells and TG(+) HEC cells displayed higher proliferation, migration and angiogenesis ability than that of human HEC-I cells and TG(−) HEC cells. Inactivation of PP2A was observed in TG(+) HEC cells and human HEC-P cells, but not in TG(−) NEC cells and human HEC-I cells (*P* < 0.001) (Fig. 6H), and was accompanied by activated AKT and ERK phosphorylation (Fig. 6I, 6J). Moreover, similar to the observations in bEnd.3 cells, decreased association of the PP2A/B subunit and the PP2A A, C subunits was also observed in human HEC-P cells, while the heterotrimeric PP2A/A-B-C complex remained intact in human HEC-I cells (Fig. 6K). These results suggest that disruption and inactivation of the PP2A complex and the associated changes in downstream pathways are key molecular and pathway alterations in hemangioma endothelial cells.

### The binding between endoglin and the PP2A/B subunit is involved in the disruption of the PP2A complex in human hemangioma specimens

Humans are not the natural hosts of polyomavirus, suggesting that PyMT is not the natural cause of the dissociation of the PP2A/B subunit from the PP2A AC dimer in human hemangioma. Hence, we searched for the “X” factor that leads to the disruption and inactivation of PP2A in human proliferating phase hemangioma endothelial cells. Bindings between PP2A and several vascular endothelial cell-specific proteins, such as CD31, CD34, KDR and endoglin, were tested. Among these molecules, only endoglin was found to bind to and disrupt the PP2A complex.

**Figure 7:**
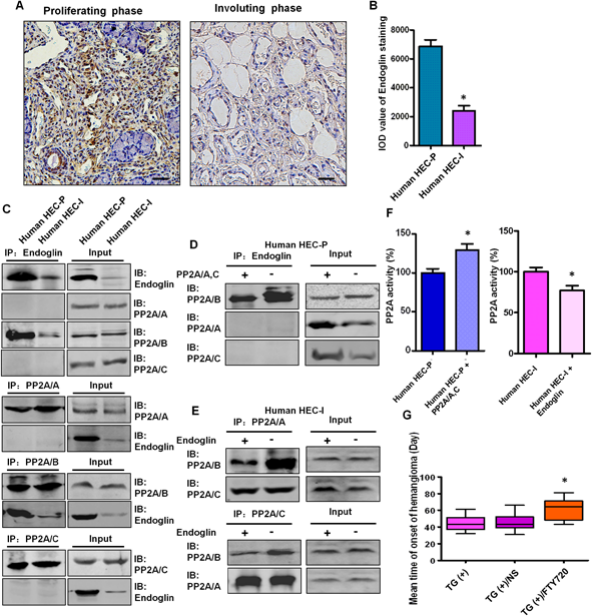
The binding between endoglin and the PP2A/B subunit is involved in the disruption of the PP2A complex in human hemangioma specimens **A.** Representative images of immunohistochemical staining of endoglin in human proliferating phase hemangioma specimens and involuting phase hemangioma specimens. Bar = 50 μm **B.** Quantitative analysis of the IOD value of endoglin staining. (*n* = 36, *t* test) *P < 0.05 **C.** Binding between endoglin and the PP2A/B subunit in HEC-P cells and HEC-I cells was determined by immunoprecipitation and immunoblot. **D.** Human HEC-P cells were transiently transfected with the PP2A/A and C plasmids and cell lysates were subjected to immunoprecipitation with endoglin and probed with anti-PP2A/A, B, C antibody. Competition assay results showed that ectopic expression of the PP2A/A, C subunits abolished the endoglin-PP2A/B binding. **E.** Human HEC-I cells were transiently transfected with the endoglin expression plasmids and cell lysates were subjected to immunoprecipitation with PP2A/A, C and probed with anti-PP2A/A, B, C antibody. Competition assay results showed that ectopic expression of endoglin abolished the PP2A/B -PP2A/A, C binding. **F.** Phosphatase activity assay results showed that increased PP2A activity was detected when the PP2A/A, C subunits were overexpressed in HEC-P cells. In contrast, PP2A activity was decreased when endoglin was overexpressed in HEC-I cells. (*n* = 3/group, t test) *P < 0.05 **G.** There was a significant difference in the tumor-free time between the control PyMT TG(+) mice and TG(+) mice treated with PP2A activator FTY720. (*n* = 7/group, one-way ANOVA) *P < 0.05

Endoglin is predominantly expressed in proliferating endothelial cells and represents a specific marker of neovascularization. Strong expression of Endoglin was detected in all 26 proliferating phase hemangioma specimens, and endoglin staining was decreased dramatically in the 10 involuting phase hemangioma specimens (Fig. 7A, 7B).

As shown in Fig. 7C, binding between endoglin and the PP2A/B subunit was observed in human HEC-P cells, and this binding was relatively weak in human HEC-I cells. In the competition assay, ectopic expression of the PP2A/A and C subunits abolished the endoglin-PP2A/B binding (Fig. 7D) and increased PP2A activity in human HEC-P cells (*P* = 0.0036) (Fig. 7F), and ectopic expression of endoglin decreased the PP2A/B-PP2A/A, C binding (Fig. 7E) and decreased PP2A activity in human HEC-I cells (*P* = 0.0417) (Fig. 7F). This result indicates that endoglin can compete with the PP2A/A, C subunits for binding to the PP2A/B subunit.

### Effect of FTY720 on inhibiting hemangioma formation in transgenic mice

Our observation that the inactivation of PP2A promotes hemangioma formation prompted us to investigate the therapeutic effect of a PP2A activator on this disease. There was a significant difference in the tumor-free time (*P* = 0.0031) observed between control PyMT transgenic mice (45.1 ± 9.6 days) and PyMT transgenic mice treated with FTY720, a PP2A activator (64.3 ± 11.4 days) (Fig. 7G), revealing a previously unknown therapeutic effect of this drug.

## DISCUSSION

Hemangioma is a benign type of tumor derived from strongly over-proliferative endothelial cell growth. The pathogenesis of the abnormal angiogenesis of hemangiomas is still unclear. In this study, we generated transgenic mice overexpressing PyMT under the control of the endothelial-specific Tie2 promoter/enhancer [5, 6]. The conditional transgenic Tie2/PyMT mice displayed a hemangioma phenotype and sustained fertility in adult mice due to avoiding embryonic lethality problems. Employing this model, we investigated the underlying generative molecular mechanism of hemangioma.

Our results showed that expression of the middle T antigen in endothelial cells can rapidly induce hemangiomas in animals, which suggests that PyMT can trigger the formation of hemangiomas in a direct manner. It has been demonstrated that the T antigen mediates many of its functions through binding to important cellular factors [7]. Therefore, we focused on the bindings between PyMT and its associated proteins to identify the mechanism through which PyMT induces hemangioma. In the present study, a specific binding between PyMT and PP2A was observed in PyMT-expressing endothelial cells. The PyMT protein was only found to bind to the AC core dimer, and not the regulatory B subunit of PP2A. Meanwhile, the dissociation of the PP2A/B subunit from the PP2A AC core dimer was observed, indicating that PyMT is able to competitively displace the PP2A/B subunit from the PP2A complex. Our findings not only demonstrate distinct patterns of binding between PyMT and PP2A in vascular endothelial cells, but also provide a possible mechanism responsible for the PyMT hemangioma transgenic mouse model.

PP2A is one of the major Ser/Thr phosphatases implicated in the regulation of many cellular processes [14–16]. Evidence suggests that PP2A can inhibit signaling pathways related to the pathogenesis of cancer cells [17–19]. These observations implicate PP2A as a potential tumor suppressor [20, 21]. However, its role in angiogenesis and hemangioma has not been reported. Our data indicate that the primary effect of the binding between PyMT and the PP2A AC core dimer is inhibition of PP2A phosphatase activity. A significant decrease in PP2A activity was observed in PyMT-expressing cells. In contrast, PP2A activity was restored when the binding was removed in PyMT-silenced cells. Considering that the protein levels of the PP2A subunits were not altered in response to PyMT expression, the inactivation of PP2A observed in PyMT-expressing cells was due to the dissociation of PP2A/B from the PP2A complex. Our data revealed a previously unknown mechanism by which PyMT inhibits PP2A activity. The finding agrees with the known functions of the PP2A/B subunit. A major function of the B subunit is to maintain the activity of PP2A. Reports from other investigators have shown that disruption of PP2A complexes via suppression of the B subunit or dissociation of the B subunit from the AC core dimer decreases PP2A activity [22–24].

Previous observations have shown that PP2A can directly dephosphorylate AKT and ERK [25–27]. To examine the biological impacts of the inactivation of PP2A on hemangioma endothelial cells, the phosphorylation status of AKT and ERK, cell proliferation, the cell cycle, apoptosis, migration and angiogenesis were evaluated. As expected, consistent with the relative PP2A activity of the cells, high levels of the phosphorylated/active forms of AKT and ERK were detected in PyMT-expressing endothelial cell, comparing with the PyMT-deficient cells. Meanwhile, PyMT-expressing endothelial cells displayed higher proliferation, an enhanced *in vitro* migration and angiogenic ability and increased *in vivo* tumorigenesis ability. For the first time to our knowledge, our data provide evidence of an anti-proliferating effect of PP2A in vascular endothelial cells, showing that inactivation of PP2A blocks the dephosphorylation of AKT and ERK and increases their activity, which subsequently promotes the proliferation, migration and angiogenic ability of endothelial cells. The acquisition of rapid growth and angiogenic ability of endothelial cells might subsequently lead to hemangioma formation. Treatment with OA, a PP2A inhibitor [28], not only markedly reversed the PyMT silencing-induced PP2A activation but also activated AKT and ERK signaling and resulted in increases in cell growth, migration and angiogenic ability, further confirming the anti-proliferating effect of PP2A in endothelial cell biology.

We then confirmed the importance of *in vitro* cell model findings in primary human hemangioma endothelial cells and primary transgenic mouse endothelial cells. Similar to the observations in *in vitro* cell model, reduced PP2A activity and high levels of p-AKT and p-ERK expression were also found in both primary TG(+) HEC cells and human HEC-P cells compared with TG(−) NEC cells and human HEC-I cells. Simultaneously, dissociation of the B subunit from the PP2A complex was detected in human HEC-P cells, but not in human HEC-I cells. These findings demonstrate that disruption and inactivation of the PP2A complex is a common molecular event in both animal models and hemangioma patients.

Then, a question arose regarding the fact that polyomavirus is a murine virus, and humans are not the natural host for polyomavirus. Therefore, in human hemangioma patients, some other factor may disrupt the heterotrimeric PP2A holoenzyme and, in turn, inhibit PP2A activity, which functions similarly to PyMT in mice. Considering that disruption of the PP2A complex was only observed in human proliferating hemangioma endothelial cells, but not in involuting endothelial cells, it is reasonable to speculate that this factor is a specific molecule expressed in proliferating hemangioma endothelial cells. We then tested the bindings between PP2A and several proliferating vascular endothelial cell markers, such as CD31, CD34, KDR and endoglin to identify the “X” factor. It was observed that only endoglin could bind to the PP2A subunits. Unlike pan-endothelial cell markers, endoglin is reported to be more specific for newly formed endothelial cells and represents a specific marker of neovascularization [29, 30]. Endoglin was found to compete with the PP2A/A, C subunits for binding to the PP2A/B subunit. These results imply a possible role of endoglin in hemangioma initiation, such that, under certain conditions, overexpression of endoglin in early-stage hemangioma endothelial cells may disrupt the PP2A complex and reduce PP2A activity, in turn prompting endothelial cell growth, migration and angiogenesis by activating AKT and ERK. While, in the revoluting phase of hemangioma, the decreased level of endoglin abolishes its binding to PP2A, resulting in restoration of PP2A activity and the regression of hemangioma. This may also explain why the lack of a revoluting phase in PyMT transgenic hemangioma mice, because constitutively expression of PyMT causes continuous suppression of PP2A activity as well as disease progression.

At present, the chemical treatment of hemangioma remains largely empirical [31]. Although some drugs, such as rapamycin and propranolol, appear a therapeutic effect on hemangioma, the underlying mechanism is still unclear [32, 33]. Our data demonstrate an anti-proliferation and anti-angiogenesis role of PP2A in endothelial cell during the formation of hemangioma, which provides support for the development of PP2A-targeted molecular therapies. Hence, the efficacy of FTY720, a PP2A activator, regarding the treatment of hemangioma was assessed. FTY720 was originally approved by the FDA as an immunosuppressive drug [34]. Recently, this drug has been shown to exert certain anti-cancer effects via the activation of PP2A [35]. However, there are no available reports concerning the curative effect of FTY720 on hemangioma. In the present study, it was shown that treatment with FTY720 significantly delayed the occurrence of hemangioma in PyMT transgenic mice. This finding supports our previous results and indicates that restoration of PP2A represents a potential therapeutic strategy for this disease.

**Figure 8:**
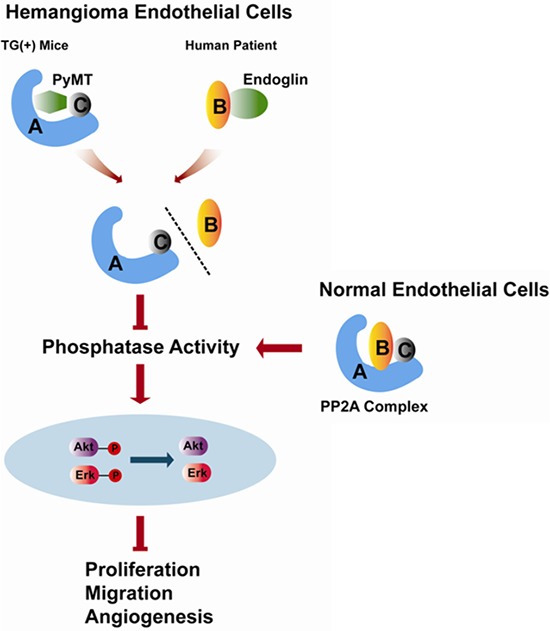
Schematic representation of the role of PP2A in regulating the biological behaviors of hemangioma endothelial cells identified in the present study

In summary, our study reveals a previously unreported anti-proliferation and anti-angiogenesis effect of PP2A in vascular endothelial cells. The inactivation of PP2A caused by dissociation of the B subunit from the PP2A core dimer and the subsequent activation of AKT and ERK promote hemangiomagenesis (Fig. 8). In addition, many studies have shown that, apart from endothelial cells, PyMT can transform epithelial cells leading to mammary tumors and other neoplasms of epithelial origin [36, 37], which impels us to think whether the PyMT-induced disruption and inactivation of the PP2A promotes tumorogenesis in these cases. Therefore, further studies are needed to confirm this mechanism in different types of tumors.

## MATERIALS AND METHODS

### Cells culture and DNA transfection

The bEnd.3 cell line was purchased from the American Type Culture Collection (ATCC) and maintained in Dulbecco's Modified Eagle's Medium (DMEM) supplemented with 10% FBS, penicillin (100 U/ml) and streptomycin (100 mg/ml) at 37°C under 5% CO_2_ [38]. PP2A/A, B, C subunit and endoglin expression plasmids were purchased from Shanghai Genechem Co. For transient transfection, the cells were transfected with the corresponding plasmid using the Lipofectamine™2000 (Invitrogen, Carlsbad, CA, USA) reagent according to the manufacturer's instructions.

### Animals

C57BL/6J mice and nude mice were obtained from the Shanghai Research Center for Model Organisms. All animal experiments were performed according to the standards of animal care outlined in the Guide for the Care and Use of Experimental Animals of the Medical College of Shanghai Jiao Tong University.

### Chemicals and treatment

The PP2A inhibitor okadaic acid (OA) and the PP2A activator, FTY720 were purchased from Sigma-Aldrich (Louis, MO, USA). OA was prepared at a final concentration of 15 nmol/L in cell culture medium. For the pharmaceutical intervention experiment, four-week-old PyMT transgenic mice (*n* = 7/group) were treated with FTY720 (5/mg/kg/d) or vehicle (normal saline, NS) via oral gavage for 10 days.

### Cloning, transgene construction and production of transgenic mice

The pPyMT1 plasmid, containing the full-length of PyMT gene, was obtained from ATCC. The Tie2 promoter and enhancer were isolated via PCR from C57BL/6J mouse genomic DNA, with KpnI and SalI ends or ClaI and SalI ends, respectively. A Kozak consensus sequence and PyMT DNA was PCR amplified with XhoI and NheI ends. A junction fragment with XbaI and ClaI ends was amplified using the pGL3 Basic plasmid (Promega, Madison, WI, USA) as a template (all PCR primers are listed in Supplemental Table S1). Then, the Tie2 promoter, PyMT cDNA, junction fragment and Tie2 enhancer segments were sequentially subcloned into the pGL3 Basic vector between the KpnI and SalI sites. This modified plasmid is referred to as pTie2-PyMT. The Tie2/PyMT insert DNA was excised from pTie2-PyMT through PvuI digestion. Transgenic mice were generated through pronuclear microinjection of the purified DNA fragments into the male pronucleus of C57BL/6J zygotes as previously described [39]. Eggs that survived microinjection were transferred into the oviducts of pseudo-pregnant foster females. TG(+) and TG(−) mice were selected via PCR screening of genomic DNA from tail biopsies. Then, the transgenic founders were bred to produce offspring.

### Histological and immunohistochemical (IHC) examination in transgenic mice

For histological examination, sample tissues were collected from the ear mucosa, the skin of the palm, and the stomach, lung, liver, kidney, and spleen of the transgenic mice. Following standard protocols, formalin-fixed samples were paraffin embedded, sectioned, and then stained with hematoxylin and eosin. Immunohistochemistry for the examination of the PyMT, CD31, Glut-1, Ki-67, CD133, CD34, p-75, Sox-10, Oct-4, STAT-3 and vimentin was performed using standard methods. The tissue sections were incubated with a rat monoclonal PyMT antibody (Abcam) (1:100 dilution), a rabbit polyclonal CD31antibody (Abcam) (1:100 dilution), a rabbit polyclonal Glut-1 antibody (Abcam) (1:100 dilution), a rabbit polyclonal Ki-67 antibody (Abcam) (1:200 dilution), a rabbit polyclonal CD133 antibody (Proteintech) (1:100 dilution), a rabbit monoclonal CD34 antibody (Abcam) (1:50 dilution), a rabbit polyclonal p-75 antibody (Abcam) (1:50 dilution), a rabbit polyclonal Sox-10 antibody (Abcam) (1:50 dilution), a rabbit monoclonal Oct-4 antibody (Abcam) (1:50 dilution), a goat polyclonal STAT-3 antibody (Abcam) (1:50 dilution) or a rabbit monoclonal vimentin antibody (Abcam) (1:50 dilution) overnight at 4°C. The bound antibody was detected with a goat anti-rat or goat anti-rabbit secondary biotinylated antibody for 30 min at room temperature (Abcam) (1:200 dilution) and visualized using diaminobenzidine as achromogenic substrate. The sections were then counter stained with hematoxylin.

### Tissue specimens from hemangiomas and IHC examination

Specimens were obtained from 26 proliferating phase hemangiomas (patients aged 3 to 14 months) and from 10 involuting phase hemangiomas (patients aged 2 to 6 years) patients who underwent surgical resection between 2000 and 2014 in the Department of Oral and Maxillofacial Surgery. The clinical diagnosis was confirmed through histological analyses performed in the Department of Oral Pathology. The IHC analysis of endoglin (Abcam) (1:100 dilution) was performed according to the method described previously.

The level of IHC staining was quantified using a semiautomated computerized image analysis system (Image Pro Plus 6.0; Media Cybernetics, USA) [40].

Integrated optical density (IOD) of positive staining was calculated for each tissue section.

### Isolation of hemangioma endothelial cells

Human HEC-P cells, human HEC-I cells, and TG(+) HEC cells were isolated from human proliferating phase hemangioma specimens, involuting phase hemangioma specimens and PyMT transgene-positive mice, respectively based on the previously described methods [41, 42]. Briefly, hemangioma tissues were cut into small pieces, which were first digested in DMEM containing 0.2% collagenase for 15 min at 37°C and then in 0.1% trypsin/0.1% ethylene diamine tetraacetic acid (EDTA) for another 5 min. The mixtures were subsequently centrifuged at 500 g for 5 min. The pellet was resuspended and seeded in 2% gelatin-precoated flasks and incubated at 37°C. TG(−) NEC cells were isolated from transgene-negative mice and then separated and purified via enzymatic digestion and density gradient centrifugation as previously reported [43]. Briefly, cerebral cortices devoid of cerebella, white matter, and leptomeninges were cut into small pieces and homogenized. The homogenates were suspended and centrifuged. The pellet was digested in 0.1% collagenase/dispase for 6 h at 37°C. Then, the digested microvessels and individual endothelial cells were subsequently seeded in 2% gelatin-precoated flasks and incubated at 37°C.

### Lentiviral shRNA knockdown of PyMT in bEND.3 cells

Stable PyMT silencing in bEnd.3 cell lines (bEnd.3 PyMT Si) was achieved using RNA interference. Two PyMT small interfering RNA (siRNA) sequences (PyMT S1: GGAAGAATGCAGCAGGCATAT, PyMT S2: GGTGGAAGCCATGCCTTAATG) were designed using the software siRNA Target Designer. Knockdown of the PyMT gene in bEND.3 cells was performed using an shRNA lentivirus (GeneChem, Shanghai, China). Two stable cell lines with down-regulated expression of PyMT(designated bEnd.3 PyMT S1 and bEnd.3 PyMT S2) were employed for further experiments. Cells transfected with the viral vector containing scrambled shRNA (GCACTACCAGAGCTAACTCAGATAGTACT) were used as the negative control (designated bEnd.3 NC).

### Tumorigenesis assay

Cells (2×10^6^) from the bEnd.3 parental line, bEnd.3 NC cells, and bEnd.3 PyMT S1 cells and bnd.3 PyMT S2 cells were injected subcutaneously on each side into the rear flanks of 6-week-old male nude mice (two sites per mouse and five mice per cell line). Tumor sizes were monitored with calipers twice a week. At the end of the experiment, mice were euthanized by cervical dislocation. The tumor volume (cm^3^) was calculated as (L × W^2^)/2, where L = length (cm) and W = width (cm).

### Immunoprecipitation and immunoblotting

Cells were lysed in a buffer containing 50 mmol/L Tris-HCl, pH 7.5, 150 mmol/L NaCl, 1 mmol/L EDTA, a protease inhibitor cocktail, and 0.3% CHAPS. Cell lysates were incubated overnight at 4°C with the indicated antibodies with constant shaking prior to incubation with protein A/G magnetic beads. Then, the samples were spun down for 1 min and the pelleted beads were washed sequentially with PBS with 1% Triton-X-100, PBS with 0.5% Triton-X-100, and PBS with 0.1% Triton-X-100. The pellets were subsequently re-suspended in SDS sample buffer and subjected to Western blotting. The antibodies used in these experiments included PP2A/A (Millipore, Darmstadt, Germany), PP2A/B (Millipore), PP2A/B’ (Millipore), PP2A/C (Millipore), PyMT (Abcam), endoglin (Abcam), ERK 1/2(Abcam), phospho-ERK 1/2 (Abcam), AKT (Abcam) and phospho-AKT (Abcam).

### Competition assay

bEnd.3 cells, human HEC-P cells or human HEC-I cells were transiently transfected with the PP2A/B subunit, PP2A/A and PP2A/C subunit expression plasmids or endoglin expression plasmid, and the associations between PyMT and the PP2A subunits, endoglin and the PP2A subunits or PP2A/A, C and PP2A/B subunits were then examined via IP-Western blotting.

### Phosphatase activity assay

Cells were washed with pre-chilled PBS once and incubated with phosphatase lysis buffer on ice for 10 min. Then, the cells were collected and centrifuged at 2 000 g for 5 min at 4°C. The supernatant was collected, and the protein concentration was measured. PP2A activity was determined using a PP2A immunoprecipitation phosphatase assay kit (Millipore), following the manufacturer's protocol.

### Proliferation assay

The MTT (3-[4, 5-dimethylthiazol-2-yl]-2, 5-diphenyltetrazoliumbromide) colorimetric assay was employed to screen for cell proliferation. Briefly, cells were seeded in 96-well plates at a density of 2 × 10^3^ cells/well. Twenty microliters of MTT (5 mg/ml) was added to each well, and cell culture was continued for 4 h. After aspiration of the medium, the cells were lysed with DMSO. The absorbance was measured using a microplate reader at a wavelength of 490 nm. These measurements were carried out for 6 consecutive days, and the cell growth curve was plotted. The experiment was performed in triplicate.

### Apoptosis detection assay

The Annexin V-FITC/PI Apoptosis Detection Kit (BD Biosciences, San Jose, CA, USA) was used following the manufacturer's instructions. Cells showing Annexin V+/PI− staining were considered early apoptotic cells, and those showing Annexin V+/PI+ staining were considered late apoptotic cells. After the staining, cells were immediately analyzed using a BD FACS Calibur flow cytometer and the CELL Quest software. The experiment was performed in triplicate.

### Cell cycle analysis

Cells were collected and fixed in 70% ethanol, washed two times with ice-cold PBS and resuspended in 500 μL of PBS. Cell suspensions were incubated with RNase A (50 μg/ml) for 30 min at 37°C and sequentially stained with PI (50 μg/ml) for 1 h and analyzed via flow cytometry. Three independent experiments were performed.

### Transwell migration assay

Cell migration was evaluated using 24-well Transwell units with 8 μm-porosity polycarbonate filters. A 200 μl aliquot of serum-starved cells (5 × 10^5^ cells/ml) was added to the upper polycarbonate membrane insert, and 600 μl of cultured medium with 10% fetal bovine serum was added to the lower chamber. After 24 h of incubation, the membranes were fixed with 5% glutaraldehyde in PBS for 10 min and then stained with 0.5% toluidine blue. The number of migratory cells was counted five times in random fields under a microscope. Experiments were performed in triplicate.

### *In vitro* angiogenesis tube formation assay

Matrigel (10 mg/ml, BD Biosciences) was plated into flat-bottomed 6-well tissue culture plates and incubated at 37°C for 30 min to allow the Matrigel to polymerize. Then, 1 × 10^5^ cells were seeded into the Matrigel-coated plates. After 48 h, the plates were monitored and photographed. Results were quantified by measuring the joint numbers in the field [44]. Experiments were performed in triplicate.

### Statistics

All values are expressed as the average ± SD. Data sets were analyzed with the aid of GraphPad Prism Software, version 6 (GraphPad). Differences between groups were assessed with either 2-tailed Student's *t* tests or, in the case of multiple groups, one-way ANOVA with Tukey's multiple comparison test. *P* values of less than 0.05 were considered to be significant.

## SUPPLEMENTARY TABLE


