# Supplementary material for: Disruption and inactivation of the PP2A complex promotes the proliferation and angiogenesis of hemangioma endothelial cells through activating AKT and ERK
Source: Oncotarget. 2015 Jul 27;6(28):25660–76. doi: 10.18632/oncotarget.4705 (PMC4694857; doi:10.18632/oncotarget.4705)
Supplement: Supplementary file 1 [file oncotarget-06-25660-s001.pdf]

SUPPLEMENTARY TABLE

Supplementary Table S1. Cloning and Genotyping primers used in this study

**Cloning Primers**	
Tie2 promoter	Forward Primer<br>CATGGTACCGCGGAAGCTTACTAAGATCTAATGAAAATC<br>Reverse Primer<br>CATGTCGACTTCAACAACTCACAACTTTGCG
PyMT	Forward Primer<br>CATCTCGAGAGCCTCACCACCATCATGGATAGAGTTCTGAGCAGAG<br>Reverse Primer<br>CATGCTAGCCTAGAAATGCCGGGAACG
Junction fragment	Forward Primer<br>GTGTAATTCTAGAGTCGGGGC<br>Reverse Primer<br>CGACGGATCCTTATCGATTTTACC
Tie2 enhancer	Forward Primer<br>CATATCGATCTCGAGGTCCAGTATGGCTTC<br>Reverse Primer<br>CATGTCGACGTACCATTATTGTTTTACTTGGGAGG
Genotyping Primers	
PyMT	Forward Primer<br>CCCGAGATGTGCTGAACC<br>Reverse Primer<br>CGTGTAGTGGACTGTGGC
